# Proteins in scalp hair of preschool children

**DOI:** 10.3390/psych6010009

**Published:** 2024-01-29

**Authors:** Cynthia R. Rovnaghi, Kratika Singhal, Ryan D. Leib, Maria Xenochristou, Nima Aghaeepour, Allis S. Chien, Monica O. Ruiz, Deendayal Dinakarpandian, Kanwaljeet J. S. Anand

**Affiliations:** 1 Child Wellness Lab, Maternal & Child Health Research Institute, Stanford University School of Medicine, Stanford, CA; 2 Stanford University Mass Spectrometry (SUMS) Lab, Stanford University, Stanford, CA; 3 Departments of Anesthesiology (Research), Biomedical Data Science & Pediatrics (Neonatology), Stanford University School of Medicine, Stanford, CA; 4 Department of Medicine (Biomedical Informatics Research), Stanford University School of Medicine, Stanford, CA; 5 Departments of Pediatrics (Critical Care Medicine) and Anesthesiology (by courtesy), Stanford University School of Medicine, Stanford, CA

**Keywords:** hair biomarkers, proteomics, brain development, developmental psychology, preschool children, non-structural proteins

## Abstract

**Background.:**

Early childhood experiences have long-lasting effects on subsequent mental and physical health, education, and employment. Measurement of these effects relies on insensitive behavioral signs, subjective assessments by adult observers, neuroimaging or neurophysiological studies, or retrospective epidemiologic outcomes. Despite intensive search, the underlying mechanisms for these long-term changes in development and health status remain unknown.

**Methods.:**

We analyzed scalp hair from healthy children and their mothers using an unbiased proteomics platform using tandem mass spectrometry, ultra-performance liquid chromatography, and collision induced dissociation to reveal commonly observed hair proteins with spectral count of 3 or higher.

**Results.:**

We observed 1368 non-structural hair proteins in children, 1438 non-structural hair proteins in mothers, with 1288 proteins showing individual variability. Mothers showed higher numbers of peptide spectral matches and hair proteins compared to children, with important age-related differences between mothers and children. Age-related differences were also observed in children, with differential protein expression patterns between younger (2 years and below) and older children (3-5 years). We observed greater similarity in hair protein patterns between mothers and their biological children as compared to mothers and unrelated children. The top 5% proteins driving population variability represent biological pathways associated with brain development, immune signaling, and stress response regulation.

**Conclusion.:**

Non-structural proteins observed in scalp hair include promising biomarkers to investigate the long-term developmental changes and health status associated with early childhood experiences.

## 1. Introduction

Early human development remains exquisitely sensitive to parental, environmental, and societal influences that multiplex the history of each individual (via genetic and epigenetic factors) with their daily experiences. Variations in these factors, such as stress and social determinants of health, can singly or collectively introduce differences in developmental outcomes^1–4^. Such differences are then magnified in the higher-order cognitive and behavioral capacities of the human mind-brain-body connectome, which are built on a series of sequential or staggered developmental epochs that can enable or constrain their future potential, role(s) in society, as well as their mental and physical health^4–8^.

Objective assessment of social, emotional, or other environmental inputs across multiple timescales is challenging in early childhood. These challenges result from most subjects being pre-verbal, coming from unknown environments, or accompanied by unreliable, fearful, or distrusting historians^1, 3, 9, 10^. Developmental timescales can also range from milliseconds to minutes (e.g., affecting acute neuromodulatory tone, neuronal oscillations, neuroendocrine changes), days to weeks (e.g., affecting circadian rhythms, metabolic functions, memory and learning), or months to years (e.g., affecting brain growth and brain plasticity, or emerging cognitive, behavioral, or social capacities)^4, 11^. Neurophysiological, neuroimaging, and observational studies have attempted to describe and quantify early developmental changes, but there remains a need for non-invasive, objective biomarkers that can be measured serially across the months and years required for childhood development^12–15^.

Human scalp hair of preschool children, derived from the neuroectoderm and mesoderm, grows constantly at about 1 cm/month and evolves via prenatal lanugo, postnatal vellus, intermediate medullary, and terminal hair stages^16^. Hair contains 65-85% proteins, 15-35% water, 1-9% lipids, and 0.1-5% pigments like melanin and trace elements^17^. Constantly growing scalp hair incorporates both endogenous and exogenous proteins in a time-averaged chronological manner^18^, unlike any other biospecimens^19^. Therefore, it is used routinely to monitor drug exposures, heavy metals, and other environmental toxins^20^, or even reflect the social determinants of health^3^.

Developmentally regulated hair proteins could offer biomarker candidates for the mind-brain-body connectome with the potential to monitor health status in real-time during early childhood development. However, all published data on hair proteins are limited to adult subjects, include relatively small sample sizes, and focus mainly on structural hair proteins. Lee et al. reported 343 hair proteins from three adults, showing evidence for post-translational modifications^21^. Laatsch et al. analyzed hair from 18 males and 3 females, reporting ethnic differences in keratins and keratin-associated proteins (KAPs)^22^. Carlson et al. characterized hair proteins from one adult with limited sample availability^23^ whereas Wu et al. used hierarchical protein clustering to match 10 monozygotic twin pairs and differentiate them from unrelated individuals^24^. Parker et al. reported quantifiable measures^21^ of identity discrimination and racial ancestry by detecting genetically variant peptides in the structural hair proteins for forensic purposes^25^.

To fill the extant gaps in knowledge, we analyzed non-structural hair proteins using ultra-performance liquid chromatography-tandem mass spectrometry (UPLC-MS/MS) and ELISA based validation studies conducted on a limited subset of the detected non-structural hair proteins present in preschool children and their mothers. Our subjects were not exposed to early life adversity, as evidenced by parental income, household structure, health insurance, and parent education^5^ or by their hair cortisol concentrations (HCC)^4, 26^.

## 2. Materials and Methods

After IRB approval and parental consent, mothers and children aged 1-6 years were enrolled from local preschool facilities. All children were developmentally appropriate, healthy, and belonged to stable nuclear families (Table 1). We excluded children with tinea capitis, alopecia areata, eczema, or other scalp conditions; those receiving any prescription or over-the-counter drugs; or steroid therapy in the past 3 months; or those with chronic medical conditions, developmental delay, or chemical exposures to hair prior to study entry. Hair samples from the posterior vertex (1 cm^2^ area) were trimmed at 0.1 mm from the scalp and stored in Ziploc^®^ bags at 4°C.

### 2.1 Hair protein extraction

Proprietary methods were developed for the extraction of soluble protein components of human scalp hair.

### 2.2 Proteomics method

Protein pellets were resuspended in 50 mM ammonium bicarbonate in the presence of 0.0015% ProteaseMAX (Promega) and total protein amount was estimated with Pierce BCA assays (Thermo Fisher Scientific) for a consistent loading of all samples. Proteins were digested with 0.25 μg of Trypsin/LysC (Promega) at a 1:100 enzyme/substrate ratio overnight at 37°C. Proteolytic digestion was quenched with 1% formic acid; peptides were dried by speed vac before dissolving in 30μl of reconstitution buffer (2% acetonitrile + 0.1% Formic acid) to a concentration of 1 μg/μl; 2 μl of this solution was injected into the MS instrument.

Experiments were performed on the Orbitrap Fusion Tribrid mass spectrometer (Thermo Scientific) coupled with ACQUITY M-Class ultra-performance liquid chromatography (UPLC, Waters Corporation). For a typical LCMS experiment (Liquid Chromatography/Mass Spectrometry), a flow rate of 450 nL/min was used, where mobile phase A is 0.2% formic acid in water and mobile phase B is 0.2% formic acid in acetonitrile. Analytical columns were pulled using fused silica (I.D. 100 microns) and packed with Magic 1.8-micron 120Å UChrom C18 stationary phase (nanoLCMS Solutions) to a length of ~25 cm. Peptides were directly injected onto the analytical column using a gradient (2-45% B, followed by a high-B wash) of 80 minutes. The MS was operated in data-dependent fashion using CID (collision induced dissociation) for generating MS/MS spectra, collected in the ion trap with collisional energy set at 35.

The *.RAW data files were processed using Byonic v3.2.0 (ProteinMetrics) to infer protein isoforms using the Uniprot *homo sapiens* database. Proteolysis with Trypsin/LysC was assumed to be semi-specific allowing for N-ragged cleavage with up to 2 missed cleavage sites. Precursor mass accuracies were held within 12 ppm and 0.4 Da for MS/MS fragments. Proteins were held to a false discovery rate (FDR) of 1% or lower, using standard target-decoy approaches^27^, and only the proteins with >3 spectral counts were selected for further data processing; keratins and KAPs were removed at this stage.

### 2.3 Generation of age-associated proteomic libraries

Initially, the standard UPLC-MS/MS methods (section 2.2) were employed to identify non-structural hair shaft proteins, using protein purification to remove keratins and for establishing age-associated hair shaft proteomic libraries using pooled hair samples from 40 children of diverse race/ethnicity (Asian, White, Mixed, or Other races; Hispanic/non-Hispanic ethnicity), aged 1-5 years (mean/SD = 44.5 months±12.6 months), and 43 mothers also of diverse race/ethnicity (aged 39 years±5 years). Utilization of large numbers of individuals of diverse race and ethnicity favors our ability to detect representative patterns of non-structural proteins incorporated in the hair shaft. We observed 1368 non-structural hair proteins in children, 1438 non-structural hair proteins in mothers, with 1288 proteins showing individual variability. The total number of age-associated proteins discovered in these libraries were also detected in the analyses of 40 independent individual subjects that had not been used for generation of the libraries. Individual hair samples from 8 mothers with 16 biologically-related children and 16 unrelated children were analyzed against the pooled hair protein libraries to create a master library of hair proteins. These data were deposited through the PRIDE repository^28^ into the ProteomeXchange Consortium^29, 30^.

### 2.4 Human scalp hair shaft proteoforms validation studies

Surplus volumes of protein remaining after UPLC-MS/MS generated libraries, individual evaluations, and quantification of hair cortisol concentrations (HCC) were pooled based on low, intermediate, or high HCC values. Hair cortisol (HCC) assays were validated previously^31^. These pools were evaluated using commercially available ELISA kits, used according to manufacturer’s instructions: cortisol (ALPCO/11-CORHU-E01-SLV), arginine vasopressin (AVP, Enzo/ADI-900-017A), Cu/ZN superoxide dismutase (SOD1, Enzo/ALX-850-033), glial fibrillary acidic protein (GFAP, Bioatrik/EKU04380), and HtrA serine peptidase 2 (HTRA2, Thermoscientific/EHHTRA2).

### 2.5 Statistical analysis

Spectral counts were used to calculate Euclidean distances between individuals, and to determine hierarchical clustering. A correlation matrix with Spearman’s coefficient was also used for rank-based depiction of similarities between the individual hair proteomes.

Principal Component Analysis (PCA)^32–34^ was used to reduce dimensionality of this rich dataset. PCA is a widely used technique for analytical modeling of linear combinations of the original dimensions called principal components^34^. The largest proportion of data variance is captured by the first principal component, the second largest proportion of variance falls along the second principal component, and so on^32^. For the first five principal components from each PCA, we multiplied the loading scores of each protein by the percent variance explained by that corresponding principal component; these weighted scores were summed for each protein to give its Total Loading Score (TLS).


Weighted Score=Loading Score∗Proportion of Variance

Total Loading Score(TLS)==∑PC=15Weighted score

Based on their TLS values, top 5% proteins were selected as the main drivers of variability in hair protein expression.

Additionally, we used t-distributed stochastic neighboring embedding (tSNE), a non- linear probabilistic approach^35, 36^, to visualize proteins with non-linear similarity in high-dimensional space as neighbors in low-dimensional linear depictions. Unlike the reproducible PCA results, the probabilistic nature of tSNE can result in somewhat different results with each computation. To avoid serendipitous results, we ran each computation at least 10 times to ensure reproducibility. For each computation, the maximum number of iterations to converge was set to 1000, and perplexity set to the maximum permitted value. Statistical significance of tSNE clustering was calculated by how often a given statistic was reproduced in 1000 simulations of permuted versions of the dataset.

Boolean profiles of the hair proteins were also compared between the original dataset (each mother coupled with her own children) and 5000 simulated datasets, created by swapping mothers between families such that no mother was paired with her own children, but the two siblings remained together in all simulated datasets. Observed conservation in pairwise intra-family Manhattan distances from the original dataset could then be attributed to the similarities in hair protein expression between each mother and her children.

For the top 5% proteins in children (n=32), we averaged spectral counts for girls and boys separately, and divided the girls’ average by the boys’ average. Resulting values were converted to log-base 2. The same process was followed for spectral counts from mothers and children.

Log fold-change values of the top 5% proteins were used as input for Ingenuity Pathway Analysis (Qiagen: https://digitalinsights.qiagen.com/products/features/). We analyzed direct and indirect relationships between molecules based on experimentally observed data, restricted to human databases in the Ingenuity Knowledge Base. We used Random Forest (RF) models for both the classification (boy vs. girl, mother vs. child) and regression (age prediction) tasks, with protein concentrations as model features and individuals as samples^37^. In classification, the model output was the probability of an individual being female (sex classification) or being a mother (person classification). For regression (age prediction), the model output was the individual’s predicted age.

Results were based on a 10-fold cross-validation repeated 100 times. Members of the same family were included in the same set, i.e. either training or test sets, to avoid information leak due to familial similarities. For the age prediction, we evaluated results using the R^2^ coefficient of determination and the linear model p-value fitted on the predicted and observed data. For the classification tasks, we used area under the ROC curve (AUC) and the Wilcoxon-Mann-Whitney test, testing the null-hypothesis that one distribution is not stochastically greater than the other.

## 3. Results

### 3.1. Features of hair proteins

**Figure 1:**
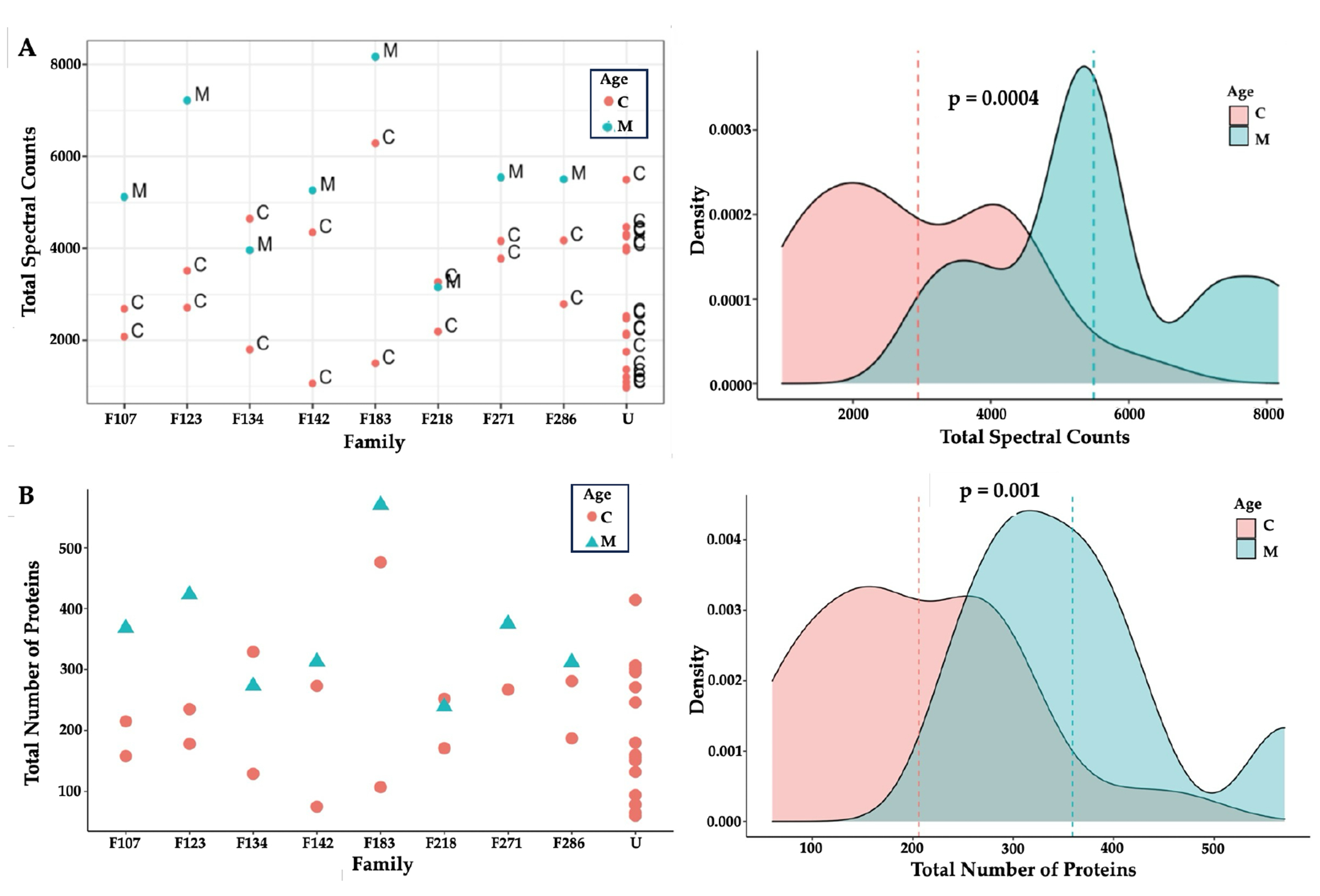
Hair Proteins in Mothers and Children **Note.** (A) Protein spectral counts (p=0.0004) and (B) the numbers of proteins observed (with spectral counts >3) were consistently higher (p=0.001, Wilcoxon tests) in the mothers (M; Cyan) as compared to children (C; Pink). Mothers and their biological children (family labels: F107, F123, F134, F142, F183, F218, F271, F288) and Unrelated children (U) are identified on the X- axis: every mother except F134 and F218 had higher spectral counts and more hair proteins than her children.

There were 3,124 proteoforms, representing the gene products of 2,278 genes. Expression of protein isoforms, alternative splicing of messenger RNA (mRNA), and post-translational modifications resulted in a higher number of hair proteins than their associated genes^21, 38^. Hair proteins observed in individual mothers and children contained 2,269 unique ‘proteoforms’ or protein isoforms; 1,438 proteins were commonly observed in mothers, 1,368 proteins were commonly observed in children, whereas 1,288 hair proteins showed individual variability among mothers and children. Higher spectral counts (p=0.0004) and higher numbers of proteins (p=0.001) were observed in mothers compared to children (Figure 1), perhaps reflecting a wider array of biological functions in adult females related to reproduction^39–41^, aging^37, 42^, or disease states^43^. These age differences were explored further in subsequent analyses.

### 3.2 Hair protein profiles in individuals and families

**Figure 2:**
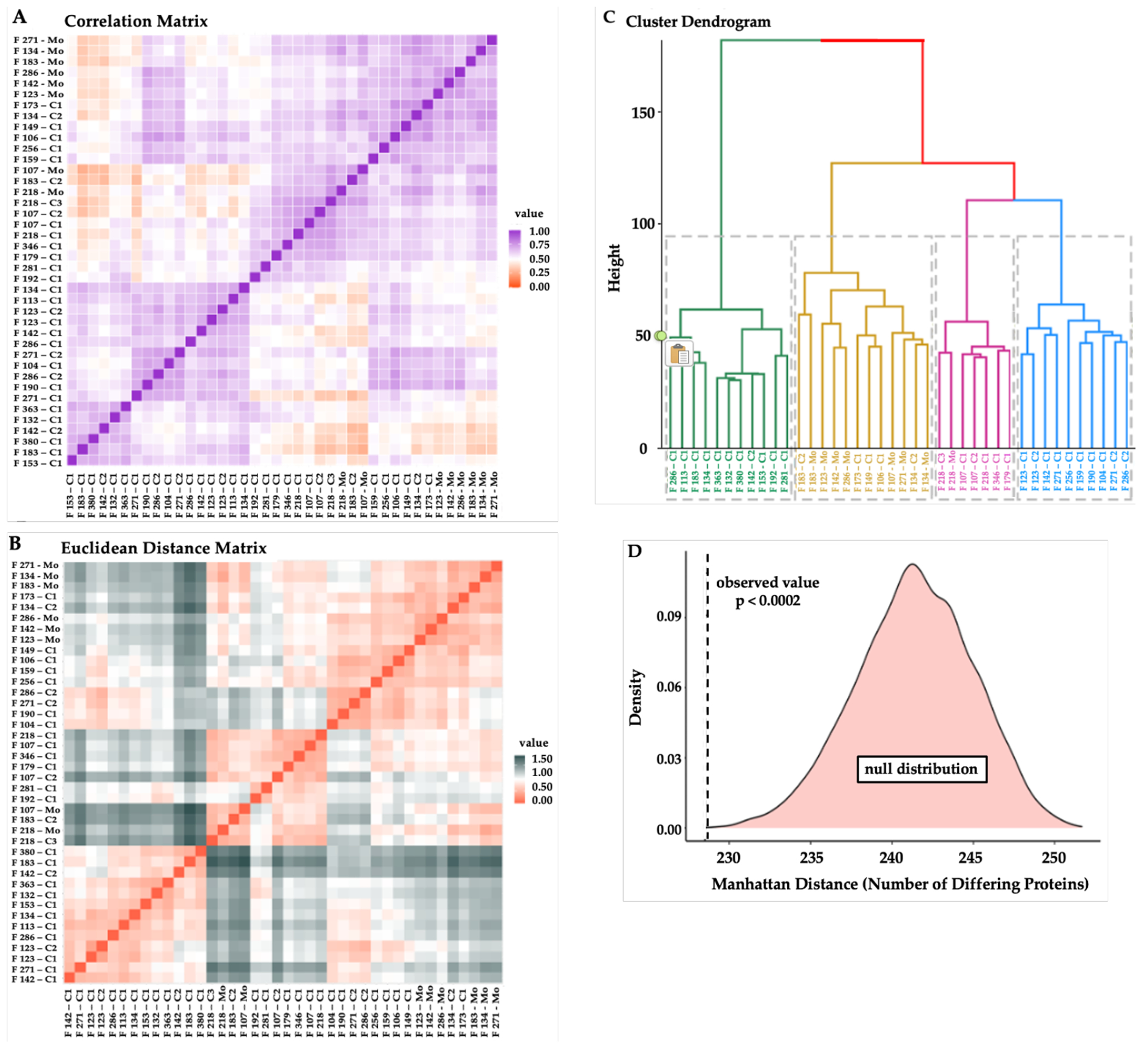
Similarities in Hair Protein Profiles of Individuals and Families **Note**. **(A)** Spearman rank correlation matrix, with high (purple) to low (orange) correlation coefficients*; **(B)** Euclidean distances based on protein spectral counts, showing individuals more closely related (red) or more distant (grey) from each other*; **(C)** Hierarchical cluster dendrogram based on log spectral counts showing 7/8 mothers grouped in one cluster (mustard) with one mother in an adjacent cluster (pink); younger children (0-2 years) in one cluster (green) whereas older children dispersed in the other clusters*; **(D)** Intra-family Manhattan distances from Boolean hair protein profiles were shorter in mothers matched with their own children (p<0.0002) vs. 5000 simulated datasets created with mismatched mothers and children. *Individuals are listed on the X- and Y-axes with their family identifier, with Mo for mother, C1 for the younger child, and C2 for the older child in each family.

Peptide spectral matches for each protein were combined to compare protein expression for all individuals and assess Spearman rank correlations. Hair proteins from the mothers were closely correlated with each other, whereas hair proteins in children showed correlations based on age and sex (Figure 2A). Euclidean distances were calculated for pairwise comparisons between individuals (Figure 2B) and used for hierarchical clustering to identify subjects with similarities in the hair protein patterns (Figure 2C). Consistent with the correlation matrix, all mothers were clustered close together, younger children (0-2 years) were mostly located in one cluster, whereas older children were clustered with the mothers (Figure 2C). Boolean profiles of the hair proteins for each mother and her two biological children showed significantly shorter intra-family Manhattan distances (p<0.0002) as compared to 5000 ‘simulated’ families with mismatched mothers and children (Figure 2D), revealing hereditary vs. environmental conservation of hair protein profiles within each family.

### 3.3. Age- and sex-related differences in hair proteins

Both PCA^32–34^ and tSNE^35, 36^ were used to reduce the data dimensionality and to identify the major contributors of hair protein variability. Principal components 1-5 accounted for 61.6% of hair protein variability for all subjects, 57.5% for all children, 84.0% for all mothers, 60.8% for mothers and related children, and 62.3% for mothers and unrelated children.

**Figure 3:**
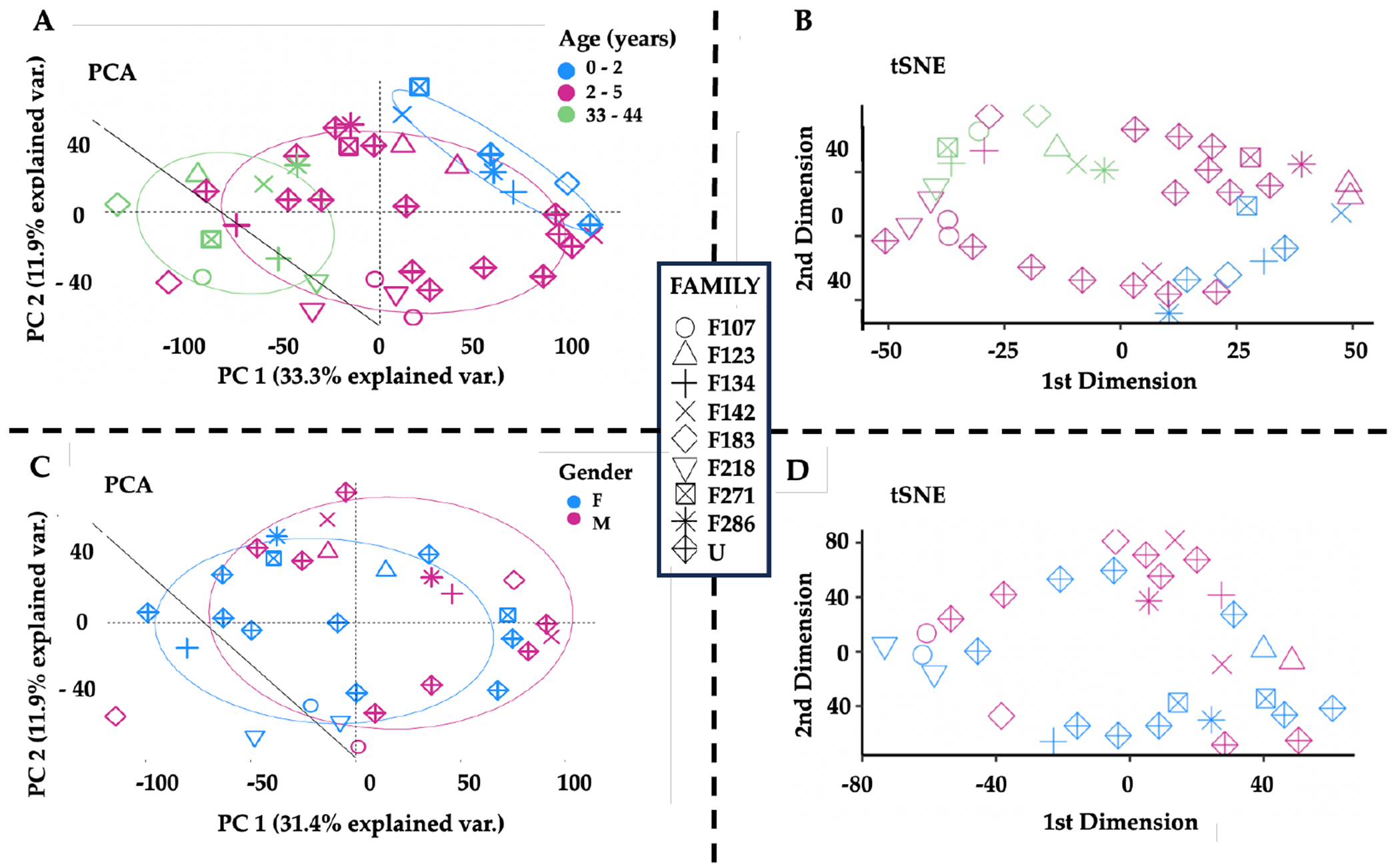
Age and Sex-related Differences in Hair Proteins **Note**. **(A)** The first two principal components showing spatial separations by age, with children above 2 years (pink) located in between the children 0-2 years (blue, upper right) and the mothers (green, lower left). **(B)** The first two tSNE dimensions by age, showing mothers in the left upper quadrant separate from the children. Higher spectral counts for 7/17 hair proteins occurred in mothers (SERPINB4, POF1B, PLEC, A2ML1, HIST1H3A, UQCRQ, AHCY) and one protein (SCGB2A1) in children (Kruskal-Wallis ANOVA and *post hoc* Benjamini-Hochberg corrections. **(C)** PCA analyses of all children showing overlapping circles for girls (blue) and boys (pink). **(D)** tSNE dimensions by sex, showing overlap between boys and girls. Higher spectral counts were observed for CSN2 (Casein beta) in boys (p=0.0184) and ALMS1 (Alström syndrome protein 1) in girls (p=0.0214) (**see** Table 3).

Age differences were observed by plotting the first two principal components (PC1, PC2) and tSNE dimensions (Figure 3). We observed two separate clusters of the younger children and the mothers, with the older children dispersed across these groups (Figure 3A). Similar clusters were observed from the remaining principal components. The tSNE projections also showed mothers located separately from the children (Figure 3B). Proteins driving these differences showed higher spectral counts in mothers vs. children for SERPINB4 (serine protease inhibitor), POF1B (actin filament binder), PLEC (cytoskeleton binding protein), A2ML1 (*α*2-macroglobulin-like proteinase inhibitor), HIST1H3A (histone), UQCRQ (electron transfer from ubiquinol to cytochrome C), and AHCY (adenosylhomocysteine hydrolase). In contrast, mammaglobin-B (SCGB2A1), a heterodimerization protein that binds androgen and other steroids, was observed only in children (Table 2). Older children had higher spectral counts for PLEC (plectin), EIF3A (eukaryotic translation initiation factor 3), AHCY (adenosylhomocysteinase), HAL (histidine ammonia- lyase), and TUBA1C (Tubulin alpha 1c), whereas younger children had higher protein spectral counts for SCGB2A1 (secretoglobin 2A member 1) and CSN2 (casein beta) (Table 3).

Sex differences showed slightly higher spectral counts in girls vs. boys (p=0.038) but no difference in the number of proteins (Table 1). PCA analyses and tSNE projections showed overlapping clusters of boys and girls (Figure 3C, 3D). When comparing individual proteins, higher spectral counts were observed for CSN2 (Casein beta, p = 0.0184) in boys and ALMS1 (Alström syndrome protein 1, p = 0.0214) in girls (Table 3).

**Figure 4:**
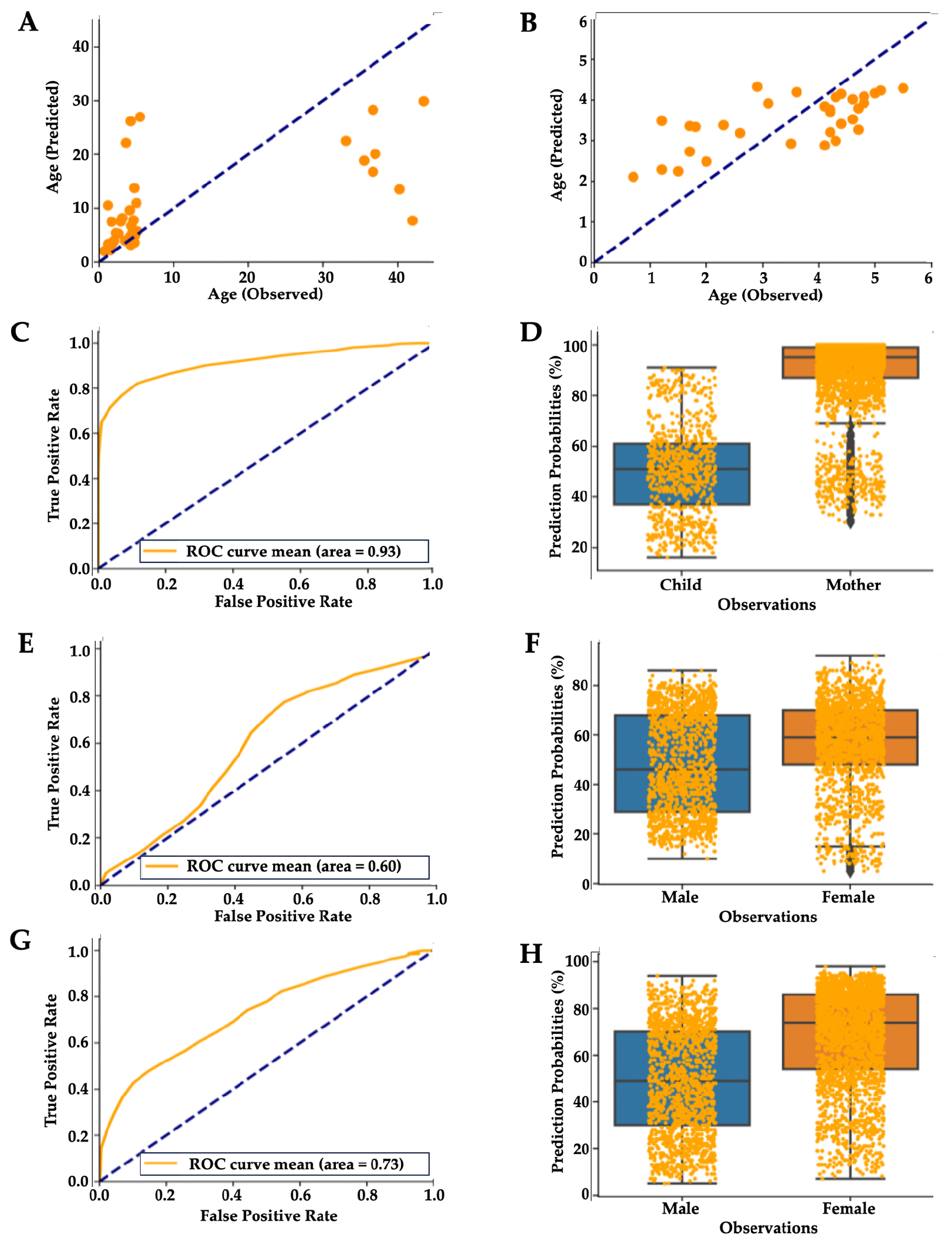
Machine Learning Algorithms Predict Age and Sex from Hair Proteins **Note**. Mean scatterplot from 100 runs of Random Forest regression showing **(A)** observed vs. predicted age for mothers and children (R^2^ 0.37, p=0.00005) and **(B)** only for children (R^2^ 0.45, p=0.00004). **(C, D)** Random Forest plot showing mean accuracy for classifying mothers and children based on hair proteins (mean area under the ROC curve = 0.93, Wilcoxon test p=0.00011). **(E, F)** Random Forest plot showing mean accuracy for classifying by sex based on hair proteins for children (mean area under the ROC curve = 0.60, Wilcoxon test p=0.1703). **(G, H)** Random Forest plot improved when classifying all participants including mothers and children (area under the ROC curve = 0.73, Wilcoxon test p = 0.00831).

To further characterize the effects of early childhood and adulthood on hair proteins, Random Forest regressions^44^ were used to predict the participants’ age from their hair protein profiles. This model predicted age differences in mothers and children (R^2^=0.37, Figure 4A), but the regression model improved (R^2^=0.45) when mothers were removed from this analysis and only children were included in this predictive model (Figure 4B). Random Forest classifier algorithms showed an acceptable mean accuracy for classifying mothers and children based on their predicted vs. observed age (mean area under the ROC curve = 0.93, Figure 4C; Wilcoxon test p=0.00011, Figure 4).

A Random Forest classifier to predict sex from hair protein profiles in children could not reliably differentiate boys from girls (mean area under the ROC curve = 0.6, Figure 4E; Wilcoxon test p = 0.1703; Figure 4F), but predictions improved when classifying all participants including mothers and children (area under the ROC curve = 0.73, Figure 4G; Wilcoxon test p = 0.0083, Figure 4H). The latter result is likely due to the age-based distinction between mothers and children, although sample size-related effects cannot be ruled out (25 vs. 17 females).

### 3.4. Top contributors to hair protein variability

**Figure 5:**
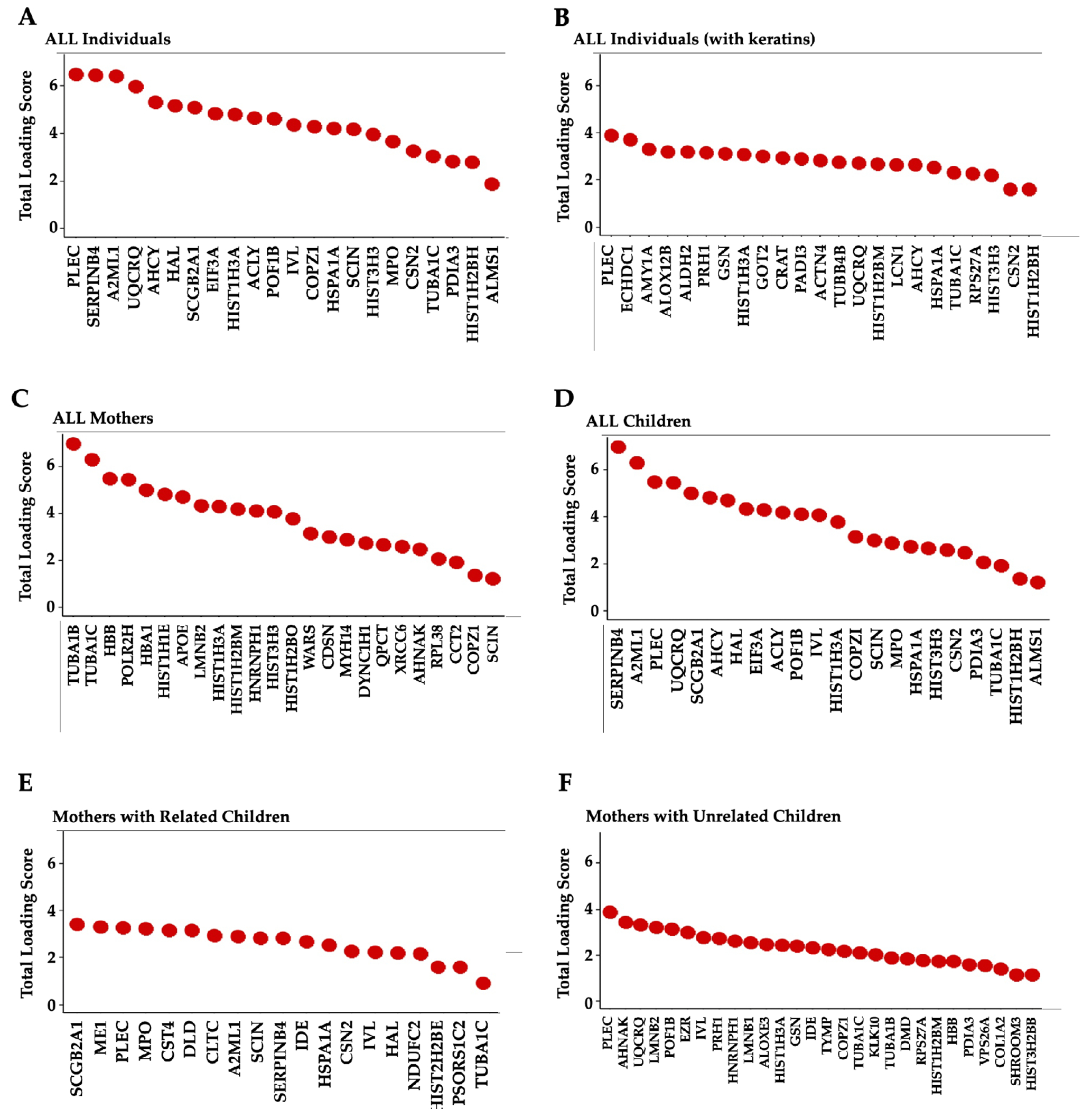
Top 5% Proteins Contributing to Hair Protein Variability **Note**. The loading scores for each protein were weighted by the percent variance explained by the corresponding Principal Component and then summed to give the Total Loading Score (TLS) for each protein. The top 5% proteins based on their TLS were identified as the most prominent contributors in each group. **(A)** All individuals (N=40, 49% of hair protein variability), **(B)** All individuals including keratins and KAPs (N=40, 64.3% variability); **(C)** All mothers (n=8, 89.5% variability); **(D)** All children (n=32, 57.5% variability); **(E)** Mothers (n=8) and their biological children (n=16) (49.3% variability), and **(F)** Mothers (n=8) and unrelated children (n=16) (64.6% variability).

The top 5% proteins identified as the most prominent contributors, based on their total loading scores (TLS), explained 64.3% of hair protein variability in all individuals, 89.5% in all mothers, 57.5% in all children, 49.3% in mothers and related children, and 64.6% in mothers and unrelated children (Figure 5). Higher TLS indicates higher influence of that protein on total variability. Keratins and KAPs are structural components, but are usually considered as contaminants in most proteomics experiments, due to their high abundance in common lab analyses. We therefore performed PCA analyses for all individuals with (Figure 5A) and without excluding the keratins and KAPs (Figure 5B). Structural proteins contributed to hair protein variability but have limited biological significance. Separate PCA analyses performed to characterize the hair proteins observed in mothers (Figure 5C), children (Figure 5D), mothers and related children (Figure 5E), and mothers and unrelated children (Figure 5F) showed the same proteins as those ranked in all individuals and all children. Other than histones, no other proteins were common between mothers and children. TUBA1C, PLEC, SERPINB4, and UQCRQ were observed in multiple subgroups.

### 3.5. Biological role(s) of the strongest contributors to hair protein variability

**Figure 6:**
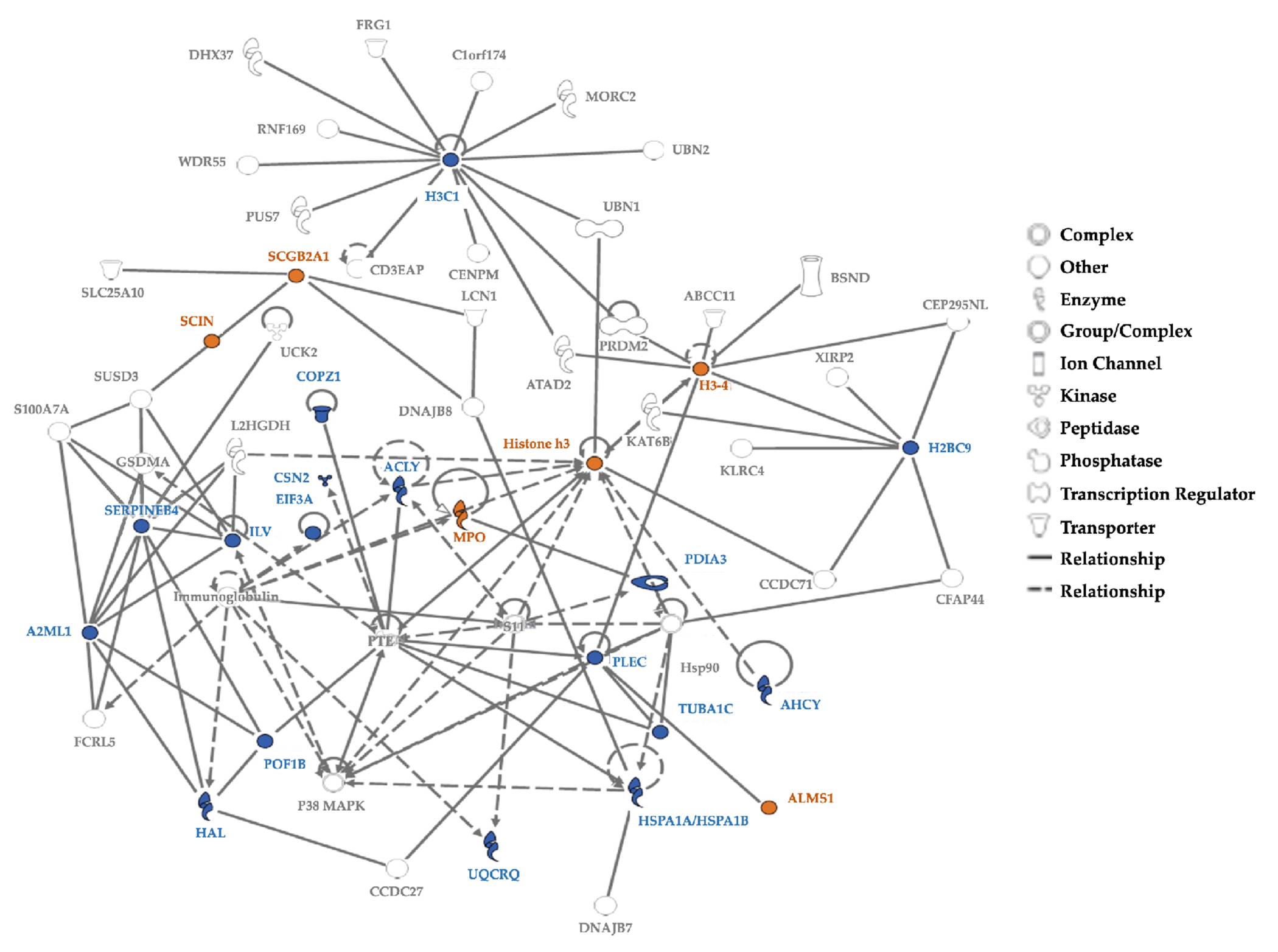
Protein Network for the Top 5% Hair Proteins Contributing to Age-related Differences between Mothers and Children **Note**. Some hair proteins had higher spectral counts in children (Orange) and others had higher spectral counts in mothers (Blue); continuous lines show direct relationships and interrupted lines denote indirect relationships. Mothers show higher spectral counts mostly for ‘enzymes’ and ‘peptidases’ involved in cellular and metabolic processes, while proteins with higher spectral counts in children belong to the ‘other’ group involved in growth and biological maturation.

**Figure 7:**
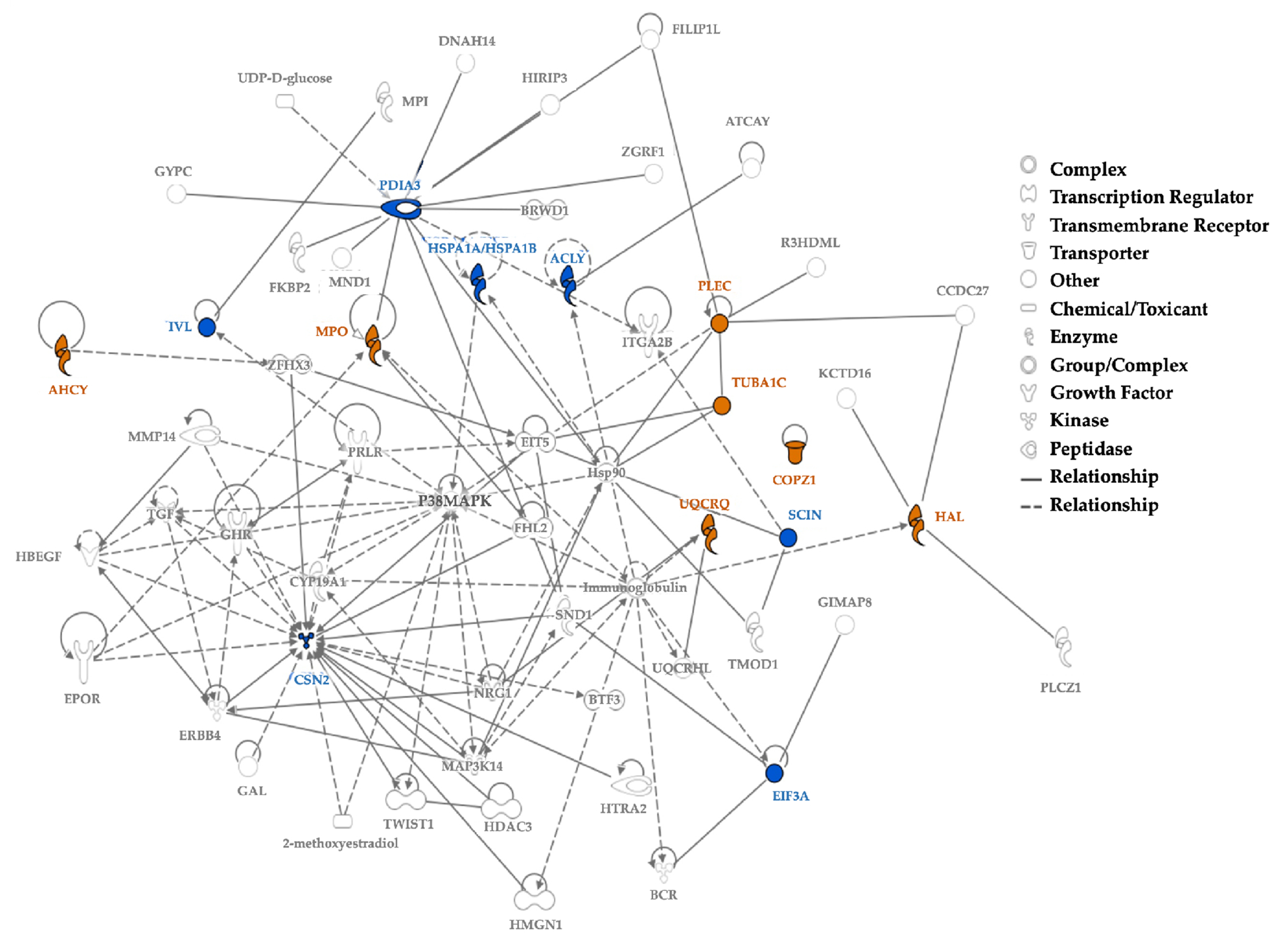
Protein Network for Top 5% Hair Proteins Contributing to Sex Differences between Boys and Girls **Note**. Protein network for top 5% hair proteins contributing to sex differences between boys and girls. Some proteins had higher spectral counts in girls (Orange) and others had higher spectral counts in boys (Blue); continuous lines show direct relationships and interrupted lines denote indirect relationships. Girls show higher protein spectral counts mostly for ‘enzymes’ or ‘ransporters’ associated with cellular localization and metabolic processes. Proteins with higher spectral count in boys are ‘enzymes’ like ‘kinases’ or ‘peptidases’ associated with biological regulation of cellular and metabolic processes.

**Figure 8:**
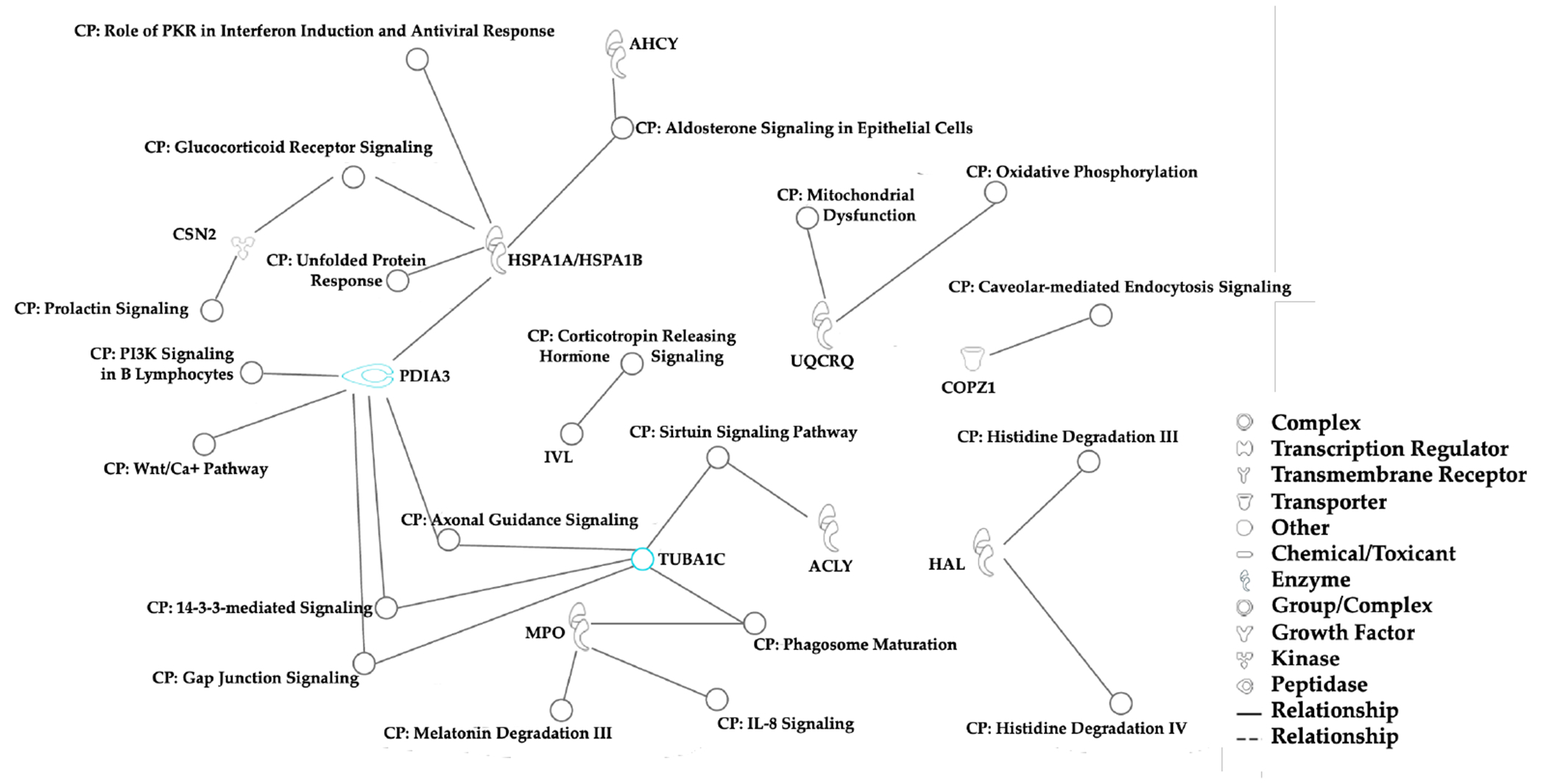
Canonical Pathways **Note**. Canonical pathways associated with biologically significant proteins from the top 5% variables in all individuals (n = 40) contributing to age- and sex-related differences were identified using the Ingenuity Pathway Analysis. Most of these proteins are involved in cellular metabolism, immune responses, brain development, and stress regulatory pathways.

Based on experimentally observed human data in the Ingenuity Knowledge Base, log- fold-change values of the top 5% proteins from our dataset were used to analyze direct and indirect relationships between protein molecules. Protein networks for the top 5% hair proteins contributing to age-related differences between mothers and children (Figure 6) and similar analyses for sex-related differences between girls and boys were examined (Figure 7). Using these molecular relationships as input for Ingenuity Pathway Analysis, we identified protein classes involved in cellular metabolism such as the protein ubiquitination pathway, Sirtuin signaling pathway, 14- 3-3 mediated signaling, Wnt-Ca^++^ pathway, histidine degradation, mitochondrial function, and oxidative phosphorylation (Figure 8). Other proteins were associated with immune responses, including phagosome maturation, IL-8 signaling, and regulation of macrophages, fibroblasts, and endothelial cells, or involved in the regulation of stress-related pathways, including corticotropin releasing hormone signaling, glucocorticoid receptor signaling, prolactin and aldosterone signaling. Finally, hair proteins associated with brain development including axonal guidance and gap junction signaling were also identified (Figure 8).

### 3.6 ELISA validation of other non-structural hair proteins

Select proteins of interest detected via standard UPLC-MS/MS methods were validated and quantified using commercially available ELISA kits. The first portion of the surplus volumes of individual protein extracts remaining after UPLC-MS/MS and HCC measures were pooled based on low, intermediate, or high HCC values. Hair sample pools were used to quantify cortisol and arginine vasopressin (AVP), which potentiate hypothalamic release of corticotropin releasing hormone^45, 46^, Cu/Zn superoxide dismutase (SOD1), an important cellular defense against reactive oxygen species^47, 48^, HTrA serine peptidase 2 (HTRA2), a mitochondrial protease chaperone that regulates cellular proteostasis and cell-signaling events^49^; and glial fibrillary acid protein (GFAP), a protein responsible for the cytoskeletal structure of glial cells^50, 51^ (Table 4).

## 4. Discussion

The chemical composition of hair^17, 52–56^ and its structural proteins (keratins, KAPs) are well-studied^22–25^, but minimal data exists on non-structural hair proteins. This study represents the first description of non-structural hair proteins in mothers and young children. We found 2,269 non-structural hair proteins with important differences between mothers and children, age- and sex-related differences among preschool children, and conserved hair protein profiles within families. Hair proteins driving variability in different populations were found to play vital roles in functions other than those of trichocytes in the hair follicle, including cellular metabolic pathways, brain development, immune signaling, and stress regulation.

We observed age-related hair protein profiles in children and mothers, with distinct patterns emerging in multiple analyses. Differences between mothers and children were largely driven by increased maternal expression of SERPINB4, PLEC, and UQCRQ. SERPINB4 is a granzyme inhibitor linked with squamous cell carcinomas and chronic liver disease^57–59^, Plectin mutations are linked with epidermolysis bullosa simplex and may be a susceptibility gene for testicular germ cell tumors^60–62^, and UQCRQ is a nuclear protein in the mitochondrial respiratory chain complex III essential for brain development^63^. Mammaglobin-B (SCGB2A1), which is linked with familial febrile seizures in preschool children^64, 65^ and chemoresistant cancers in adults^66^, was observed only in children’s hair.

We found minimal sex differences in early childhood, confirmed by Random Forest predictive models. Biological pathways for cellular metabolism and innate immunity appeared more prominent in girls, whereas brain development and stress regulation appeared more prominent in boys. Perhaps sex differences in hair proteins may be accentuated following the onset of puberty^67^. Although hair protein profiles were conserved in mothers and their biological children, future studies in mother-child dyads and monozygotic vs. dizygotic twins will be required to explore to gene × environment interactions responsible for hair protein profiles^68^.

From Ingenuity Pathway Analysis, we identified the hair proteins associated with axonal guidance^69^ and gap junction signaling^70^, both signifying important mechanisms in brain development. By cross-referencing the Uniprot database (https://www.uniprot.org/) with the Allen Brain Atlas (https://human.brain-map.org/static/brainexplorer) and the Human Brain Protein Atlas (https://www.proteinatlas.org/search/brain_category), we identified 191 hair proteins that are regionally enriched in the brain. Further studies will examine whether hair proteomics can complement neuroimaging and neurophysiological studies of early brain development^12^. A study from Nepal reported specific plasma proteins associated with higher non-verbal intelligence and pro-inflammatory proteins associated with lower intelligence in children^71^. This study, however, used an FDR of 5%, whereas the FDR threshold for our analyses was set at 1% or lower. Future developmental studies with large sample sizes could correlate hair proteins with cognitive or behavioral outcomes, thus investigating their role in brain development^72^. Thus, unbiased or targeted protein profiles from serial hair samples (or sequential hair segments in the same hair sample) could be used as probes for child development^73, 74^ or life-course studies^43, 75, 76^.

These findings must be interpreted in the light of three limitations. First, our sample size of 32 children was insufficient to examine developmental differences at each age in the preschool period. We selected healthy children from homogenous socioeconomic environments; they did not experience any adverse conditions and therefore, our data do not represent the full range of hair protein profiles present in the general population. Despite this, our sample size is larger than most other studies of hair proteomics in adults and it is the first to include mothers and children. Our study design also allowed us to investigate differences in hair protein profiles between related and unrelated individuals, as well as differences between adults and children.

Second, our proteomics platform relied on peptide spectral matches, which presented only semi- quantitative data on the abundance of hair proteins in individuals. Since this is the first study investigating non-structural proteins from hair in humans, we chose a ‘shot-gun’ proteomics approach rather than targeted and more quantitative approaches. We did, however, orthogonally confirm the presence of specific hair proteins using well-validated ELISA assays. Having established the first hair protein libraries in mothers and children, future studies can be designed for the quantitation of specific protein targets or protein groups. Lastly, we did not correlate hair proteins with the child’s developmental milestones or their cognitive and behavioral data. We feel that the sample size limitations at each age would preclude any generalizable conclusions from such analyses.

Despite these limitations, our initial findings reveal the potential importance of non-structural hair proteins as biomarkers for brain development, or other cellular regulatory pathways, providing a rich source of chronologically ordered information for life-course studies and early childhood development.

## 5. Conclusions

This research shows that exposures to family adversity, chronic stress, parenting and caregiving practices, and early attachment can be monitored by serial hair sampling to determine the child’s health status, brain development, physical and mental health. We found that hair protein profiles are related to age, sex, and family relationships. The top 5% contributors to variability in hair protein patterns are associated with regulation of: (a) **immune pathways** (for phagosome maturation, IL-8 signaling, PKR interferon induction, regulation of fibroblasts, macrophages, and endothelial cells); (b) **stress signaling pathways** (for corticotropin releasing hormone, glucocorticoid receptors, prolactin, and aldosterone); (c) **brain development** (axonal guidance, gap junction signaling); and (d) **cellular metabolic pathways** (for oxidative phosphorylation, mitochondrial dysfunction, histidine degradation, caveolar-mediated endocytosis, as well as the heat shock proteins, 14-3-3 protein, Sirtuin, and Wnt/Ca^++^ signaling pathways). When amalgamated with well-established methods for tracking changes in hair hormones, this approach may provide mechanistic explanations for the developmental sequences leading to HPA axis (dys)regulation in early life. The assessment of parent-child synchrony, the child’s circadian rhythms, or positive and negative attachments need not depend on subjective questionnaires, invasive blood sampling, or neuroimaging. We propose that non-invasive hair sampling and tandem mass spectrometry methods can be used to compare non-structural hair protein profiles in healthy, normal children against hair protein profiles in subpopulations of children with confirmed exposures to toxic stress and/or adverse living conditions. Future studies are designed to quantify and characterize panels of related hair proteins to probe changes in the immune system, stress regulation, brain development, and cellular metabolism to monitor environmental influences on the health status and development of children^2^.

## 6. Patents

Pursuant to the Patent Cooperation Treaty, an international patent was filed on November 10, 2022, identifiable in the United States Patent and Trademark Office by Application No. US2022/079619.

## Figures and Tables

**Table 1:** Demographic Characteristics and Hair Protein Data

Family Code	Subject	Age (months)	Age (years)	Gender	Race	Ethnicity	# of hair proteins	Peptide Spectral Matches (PSMs)
**F107**	Mother	450.6	37.6	F	White	Non-Hispanic	819	6533
	Child1	27.6	2.3	F	White	Non-Hispanic	568	3949
	Child2	58	4.8	M	White	Other	464	2873
**F123**	Mother	447.1	37.3	F	White	Non-Hispanic	809	10370
	Child1	24	2	F	White	Non-Hispanic	499	3728
	Child2	52.4	4.4	M	White	Non-Hispanic	573	5078
**F134**	Mother	431.8	35.9	F	White	Missing	684	5445
	Child1	20.9	1.74	M	Mixed	Missing	387	2760
	Child2	67.6	5.6	F	Mixed	Missing	759	6872
**F142**	Mother	447.3	37.3	F	Asian	Missing	650	8370
	Child1	20.1	1.7	M	Mixed	Missing	581	6208
	Child2	50.6	4.2	M	Mixed	Missing	226	2353
**F183**	Mother	530	44.2	F	Asian	Non-Hispanic	1090	10527
	Child1	8.5	0.7	M	Asian	Non-Hispanic	314	2331
	Child2	44	3.7	M	Asian	Non-Hispanic	1010	8065
**F218**	Mother	504	42	F	White	Hispanic	609	4144
	Child1	58.5	4.9	F	White	Hispanic	524	3107
	Child2	35.2	2.9	F	White	Hispanic	631	4475
**F271**	Mother	402.6	33.6	F	White	Non-Hispanic	769	7525
	Child1	15.1	1.3	F	White	Non-Hispanic	557	7161
	Child2	42.5	3.5	F	White	Non-Hispanic	600	5615
**F286**	Mother	489.8	40.8	F	White	Non-Hispanic	616	9209
	Child1	22	1.8	M	White	Non-Hispanic	403	4727
	Child2	52.5	4.4	F	White	Non-Hispanic	614	6061
**F346**	Child	50.3	4.2	M	White	Missing	475	3429
**F192**	Child	38.1	3.2	F	Other	Hispanic	283	1731
**F132**	Child	51.4	4.3	F	White	Missing	272	1892
**F363**	Child	56.6	4.7	M	Mixed	Non-Hispanic	270	1914
**F281**	Child	53.5	4.5	M	Mixed	Mixed	406	3192
**F173**	Child	51.3	4.3	F	Other	Other	835	7168
**F380**	Child	14.8	1.2	M	Asian	Missing	237	1814
**F159**	Child	62.7	5.2	F	White	Non-Hispanic	485	3830
**F179**	Child	53	4.4	F	Asian	Other	494	2926
**F149**	Child	61.3	5.1	F	Mixed	Non-Hispanic	698	5733
**F106**	Child	56.7	4.7	F	Asian	Non-Hispanic	668	6390
**F153**	Child	57.1	4.8	M	Asian	Missing	275	2549
**F256**	Child	55.8	4.7	M	Mixed	Mixed	638	7016
**F190**	Child	31.5	2.6	F	Asian	Other	527	7460
**F104**	Child	50.2	4.2	M	White	Non-Hispanic	672	8084
**F113**	Child	17.9	1.5	F	White	Non-Hispanic	441	3640

**Note**. Demographic data, total number of proteins, and peptide spectral matches observed in all 40 individuals. Mothers’ hair (n=8) had significantly higher number of proteins (p=0.001) and protein spectral matches (p=0.0004) compared to children’s hair (n=32). Related children (n=16, Cyan) are grouped with their mothers and unrelated children are listed below (n=16, Orange).

**Table 2:** Hair Proteins Mediating Differences between Mothers and Children

Entrez Gene Name	Gene Symbol: human	Expr Log Ratio	P-value	Location	Type(s)
Involucrin	IVL	−2.85	0.0576	Cytoplasm	other
Serpin family B member 4	SERPINB4	−2.452	0.0009***	Cytoplasm	other
POF1B actin binding protein	POF1B	−2.097	0.0151*	Plasma Membrane	other
Plectin	PLEC	−1.886	0.0004***	Cytoplasm	other
Alpha-2-macroglobulin like 1	A2ML1	−1.858	0.0042**	Cytoplasm	other
H3 clustered histone 1	HIST1H3A	−1.743	0.0038**	Nucleus	other
Ubiquinol-cytochrome c reductase complex III subunit VII	UQCRQ	−1.716	0.0007***	Cytoplasm	enzyme
Adenosylhomocysteinase	AHCY	−1.472	0.0040**	Cytoplasm	enzyme
Heat shock protein family A (Hsp70) member 1A	HSPA1A	−1.35	0.0569	Cytoplasm	enzyme
H2B clustered histone 9	HIST1H2BH	−1.17	0.507	Nucleus	other
Histidine ammonia-lyase	HAL	−1.087	0.0851	Cytoplasm	enzyme
COPI coat complex subunit zeta 1	COPZ1	−0.931	0.158	Cytoplasm	transporter
Eukaryotic translation initiation factor 3 subunit A	EIF3A	−0.8	0.0567	Cytoplasm	other
Tubulin alpha 1c	TUBA1C	−0.526	0.262	Cytoplasm	other
Casein beta	CSN2	−0.269	0.491	Extracellular Space	kinase
ATP citrate lyase	ACLY	−0.249	0.0954	Cytoplasm	enzyme
Protein disulfide isomerase family A member 3	PDIA3	−0.051	0.884	Cytoplasm	peptidase
Scinderin	SCIN	0.028	0.221	Cytoplasm	other
Alström syndrome protein 1, centrosome and basal body associated protein	ALMS1	0.18	0.572	Cytoplasm	other
Histone H3.4	HIST3H3	0.64	0.153	Nucleus	other
Myeloperoxidase	MPO	0.925	0.886	Cytoplasm	enzyme
Secretoglobin family 2A member 1	SCGB2A1	5.32	0.0008***	Extracellular Space	other

**Note**. Mothers showed higher spectral counts than children for 7/17 hair proteins (shaded blue), although children had higher spectral counts for SCGB2A1 (shaded orange). Of these, SCGB2A1 showed the most prominent results, with >5-fold differences from the mothers. Significance was based on Kruskal-Wallis ANOVA with post hoc Benjamini Hochberg corrections for multiple comparisons (*p-value ≤ 0.05, **p-value ≤ 0.01, ***p-value ≤ 0.001).

**Table 3:** Hair Proteins Mediating Differences between Preschool Boys and Girls

Entrez Gene Name	Gene Symbol: human	Expr Log Ratio	P-value	Location	Type(s)
Casein beta	CSN2	−3.046	0.0184*	Extracellular Space	kinase
Serpin family B member 4	SERPINB4	−1.303	0.391	Cytoplasm	other
Secretoglobin family 2A member 1	SCGB2A1	−1.036	0.0513	Extracellular Space	other
Protein disulfide isomerase family A member 3	PDIA3	−0.78	0.662	Cytoplasm	peptidase
ATP citrate lyase	ACLY	−0.531	0.585	Cytoplasm	enzyme
Myeloperoxidase	MPO	−0.493	0.581	Cytoplasm	enzyme
Involucrin	IVL	−0.476	0.804	Cytoplasm	other
Eukaryotic translation initiation factor 3 subunit A	EIF3A	−0.295	0.226	Cytoplasm	other
Alpha-2-macroglobulin like 1	A2ML1	−0.254	0.923	Cytoplasm	other
Scinderin	SCIN	−0.187	0.375	Cytoplasm	other
Heat shock protein family A (Hsp70) member 1A	HSPA1A/HSPA1B	−0.122	0.573	Cytoplasm	enzyme
POF1B actin binding protein	POF1B	0.094	0.875	Plasma Membrane	other
Histone H3.4	H3-4	0.139	0.938	Nucleus	other
Histidine ammonia-lyase	HAL	0.175	0.522	Cytoplasm	enzyme
COPI coat complex subunit zeta 1	COPZ1	0.225	0.536	Cytoplasm	transporter
Tubulin alpha 1c	TUBA1C	0.245	0.314	Cytoplasm	other
H3 clustered histone 1	H3C1	0.249	0.562	Nucleus	other
Adenosylhomocysteinase	AHCY	0.333	0.202	Cytoplasm	enzyme
Plectin	PLEC	0.441	0.256	Cytoplasm	other
Ubiquinol-cytochrome c reductase complex III subunit VII	UQCRQ	1.415	0.0976	Cytoplasm	enzyme
H2B clustered histone 9	H2BC9	1.423	0.221	Nucleus	other
Alström syndrome protein 1, centrosome and basal body associated protein	ALMS1	1.754	0.0214*	Cytoplasm	other

**Note**. Girls showed higher spectral counts than boys for several proteins (shaded orange), whereas boys had higher spectral counts for other proteins (shaded blue). CSN2 was significantly higher in boys, whereas ALMS1 was significantly higher in girls. Significance was based on Kruskal-Wallis ANOVA with post hoc Benjamini Hochberg corrections for multiple comparisons(*p-value ≤ 0.05).

**Table 4:** Validation and Correlates of Proteins Detected in Human Scalp Hair via UPLC- MS/MS

Cortisol Level Based Sample Pools Std Method	Cortisol ng/ml	AVP pg/ml	Cu/Zn SOD ng/ml	HTRA2 ng/ml	GFAP ng/ml
Low Child pool cortisol (n = 72)	40.84	14.81	0.25	7.54	0.00
Moderate Child pool cortisol (n = 21)	60.34	11.91	0.18	4.61	0.41
High Child pool cortisol (n = 7)	190.89	7.18	0.23	9.14	n/a
Low Father pool cortisol (n = 13)	22.39	8.36	0.63	9.65	2.64
Low Mother pool cortisol (n = 39)	17.24	7.88	0.49	7.71	1.45
High Mother pool cortisol (n = 7)	36.77	11.68	n/a	n/a	n/a

**Note.** Hair cortisol concentrations (HCC) were measured for 100 children and 49 parents. Groups of children and parents were determined based on low, moderate, or high HCC values. Each of the six pools of samples were loaded in duplicate on the respective ELISA plates for testing arginine vasopressin (AVP), Cu/Zn superoxide dismutase (SOD1), HTrA serine peptidase 2 (HTRA2), and glial fibrillary acid protein (GFAP). Each methods passed our criteria for low inter-assay (≤8% CV) and intra-assay (≤ 6%) variability.

## Data Availability

Minimal datasets related to results presented in this manuscript are available on request from the corresponding author. These data are not publicly available due to privacy, proprietary, and ethical considerations.
